# Anti-microbial and micro-leakage properties of orthodontic cement

**DOI:** 10.6026/9732063002001368

**Published:** 2024-10-31

**Authors:** Afreen Wahab, Chanamallappa Ganiger, Renuka Pawar, Sandesh Phaphe, Yusuf Ronad

**Affiliations:** 1 Department of Orthodontics and Dentofacial Orthopaedics, School of Dental Sciences, Krishan Vishwa Vidyapeeth (Deemed to be University), Karad, Satara, Maharashtra, India

**Keywords:** Glass Ionomer Cement (GIC), antimicrobial property, micro-leakage, N-acetylcysteine (N-AC), copper nanoparticle (Cu-NP)

## Abstract

Glass Ionomer Cement (GIC) is used for cementing orthodontic bands because of its anti-cariogenic property, which is attributed to the release of fluoride. Therefore, it is of interest to assess the antimicrobial property and micro-leakage of GIC incorporated with different concentration of N-acetylcysteine (N-AC) and copper nanoparticle (Cu-NP). Our study composed of 5 groups *i.e.* group I is control with different concentration of N-AC and Cu-NP involving each group with 8 samples. We found that, group V showed the highest zone of inhibition; while the micro-leakage was seen highest for group I with a score of 2.2 ± 1.09 and the least score was recorded for Group III (0.8± 0.37). Thus, addition of 2% Cu-NP and 15% N-AC resulted in minimal micro-leakage. We conclude that increase in concentration of N-AC and CU-NP antimicrobial property efficiency also increases; on the other hand, increase in the concentration of N-AC and CU-NP did not decrease the micro-leakage.

## Background:

Orthodontics and Dentofacial Orthopedics, is an integral discipline within the realm of dentistry, focus on the intricate interplay between dental and facial structures [1]. This specialized field is dedicated to diagnosing, preventing, and treating anomalies of tooth and jaws, recognizing the profound impact of these irregularities on both aesthetics and functions. Through the use of various orthodontic techniques, devices, and personalized treatment plans, practitioners aim to not only achieve an aesthetically pleasing smile but also to optimize the overall facial harmony [1]. Originally, removable orthodontic appliances (ROA) were employed to achieve the desired tooth movements, but such appliances cannot cause bodily tooth movements. Fixed orthodontic appliances (FOA) were introduced which involves bonding of anterior teeth and banding of posterior teeth with cements, this has since gained global acceptance (GGA). The problem with FOA is that they increase the surface area where bacteria can adhere, making it harder to maintain effective oral hygiene (OH). Thus, biofilms are more likely to develop at the tooth interface adjacent to fixed appliances (FA). This will causes more adherence of bacteria like Streptococcus mutans (SM) and Lactobacillus species(LB-S), this will lowers the oral pH below the critical level leads into formation of acids, like citric acid(CA) which will cause decalcification of enamel which eventually causes white spot lesions(WSL) observed by Zachrisson in 1977 [1]. Studies have also found that, SM and LB-S are linked to the onset and progression of white spot lesions, later leading into dental caries (DC) and periodontal diseases (PD-D) by colonizing of bacteria around the teeth. Therefore, enamel (E) decalcification (DCF) is frequently observed in areas with plaque build-up, especially in orthodontic patients [2]. White spot lesions are evident as subsurface enamel porosity (SS-EP) that manifests as chalky opacities around brackets on the tooth surface. White spot lesions are one of the most common adverse effects (AE) of FOT. The first dental cement utilized for band cementation (BC) was ZnPO4. This cement is no longer employed due to its weak adhesion (WA) and tendency to dissolve in the oral environment (OE). A significant breakthrough in dental caries was achieved by Wilson and Kent in 1972 with the invention of Glass Ionomer Cement. Studies have shown that, N-AC is a mucolytic compound having wide margin of safety as a therapeutic. This is mainly used to reduce the viscosity of mucus in pulmonary compromised patients (PCP) [3]. Nanotechnology (NT) has been utilized to enhance the effectiveness of dental materials (DM). CU-NP, in particular, have been investigated for their AB-P and ability to prevent ML. Research shows that, increased concentration of Cu-NP can enhance adhesive-dentin interfaces (ADI), improving their resistance to micro-leakage [3]. The final outcome of a FOT is to obtain the best possible esthetic along with the restoration of harmonious occlusion (HO). After de-bonding (DB), the enamel condition should be preserved close to its original form. Enamel-decalcification can cause irreversible damage and would lead to disruption of intact enamel surface [4]. Therefore, it is of interest to assess antimicrobial and micro-leakage using different concentration of N-AC and Cu-NP in GIC.

## Materials and Methods:

The current *in-vitro* study was conducted in a total 5 bottles of GIC using 3M ESPE KETAC CEM GLASS. Subsequently, they were divided into 5 groups named Group I (Conventional GIC), while experimental groups like Group II (GIC + 2% Cu-NP & 10% N-AC), Group III (GIC + 2% Cu-NP & 15% N-AC) Group IV (GIC + 2% Cu-NP & 15% N-AC) and Group V (GIC + 3% Cu-NP and 15% N-AC). For preparation of the samples, SM strain was procured, cultured with selective media and stock was prepared. Sterile Muller Hinton Agar(S-MHA) was poured into plates, with the depth of the medium set at around four milli-meters. The plates were incubated IB) for 30 minutes in an incubator to eliminate excess moisture from the surface once solidified. Isolated specimens were used for sensitivity testing. Approximately 5-6 colonies of SM-S were selected and inoculated (IO) into nutrient broth (NB) using a wire loop (WL). The broth culture (BT-C) was then incubated at 35-37° C for 2-5 hours. A sterile cotton swab (S-CS) was dipped into the diluted IO and then swabbed onto MHA-plates (PL). Excess inoculum was removed with another cotton swab. The Petri dishes (PD) were closed and left at room temperature for 5-10 min to allow the IO to dry, aiming for confluent growth. Well was prepared at seven mm diameter in each plate with the help of syringe, in order to keep distance for zone of inhibition (ZOI). Remaining agar was discarded. Disc on each well which have been inoculated was cultured with SM-S. All PL were incubated at 37°C for 24-48 hours. After incubation(IC), the area of inhibition around each well was observed as shown in (Figure 1 see PDF & Figure 2 see PDF).

The diameter (DIA) of the IH - ZOI caused by the sample against bacteria was measured. Using a digital caliper (DG-CL), the DIA-IH-ZOI (specimens + IB-Z) was measured in mm after 48 hours, and the average measurement was recorded as the day 1 value. The bacterial population usually dies due to the release of toxic metabolites if cultures were kept for a long duration. Hence, on day two, fresh agar plates (FAPL) were used and cultured, and the specimens were transferred, and IC and IN-Z was calculated. For micro-leakage evaluation, 40 PM teeth obtained from the Department of Oral and Maxillofacial Surgery (OMFS) were separated into 5 groups, with each group containing 8 teeth. The teeth underwent cleaning with pumice (PU), followed by rinsing with distilled water, and thorough drying with compressed air. Subsequently, bands were selected, pinched, and optimally adapted to the crown of each tooth. Then for each tooth band cementation was done for respective groups and then placed in distilled water for 24 hours to prevent dehydration. After cementation, the samples was subjected to 5000 thermo cycles in thermo cycling unit which would simulate six months of temperature changes in oral environment as shown in (Figure 3 see PDF).

The apices of all teeth with bands were sealed using sticky wax to prevent dye seepage, and nail polish was applied over the tooth surfaces to prevent dehydration, leaving a 1 mm gap around the bands. Afterward, all teeth were immersed in water once the nail polish had dried. To assess micro-leakage all samples were immersed in a 0.5% methylene blue solution (MBS) for 24 hours at room temperature as shown in (Figure 4 see PDF).

After removal, teeth were rinsed in tap water to remove superficial dye using a brush. Next, the samples were dried and encased in self-curing acrylic blocks (SC-AB), extending up to the occlusal surface of the bands. Using a low-speed diamond saw, the blocks were bisected in the labio-lingual direction (LL-D). Longitudinal sections from the middle portion of each tooth were cut at the occlusobuccal (OB) and occlusolingual surfaces (OL-S) as shown in (Figure 5 see PDF).

The specimens were examined under a stereomicroscope(SM) (20x magnification) to evaluate dye penetration (DP) along the cement-enamel interface (CEI). Each section was graded at both the buccal and lingual margins of the bands between the interfaces (Table 1).

## Statistical analysis:

All analysis was performed using IBM SPSS Statistics for Windows, version 26.0 (IBM Corp., Armonk, USA). Kolmogorov Smirnov and Shapiro wilk tests were applied. An inter-group comparison was conducted to compare antimicrobial activity and micro-leakage prevention among the 5 groups using Kruskal Wallis, ANOVA test followed by Mann Whitney U test for pairwise comparison. The mode of failure among the five groups was assessed using the Chi-square test.

## Results:

Table 2 shows that, the mean score of Group I is 0.1±0.00, group II is 2.63±0.16, group III is 2.19±0.13, group IV is 3.41±0.52 and group V is 4.11±0.14. The mean score is highest for group V and least for group I. Kruskal Wallis ANOVA test revealed highly statically significant difference between the groups (p = 0.000). Table 2 shows that, both the variables showed high significance in group IV i.e. 0.003 and 0.005. Table 3 shows that, the mean L-ML score of group I is 2.0±1.09, group II is 1.16±0.40, group III is 0.85±0.37, group IV is 1.83±1.32 and group V is 1.4±0.89. No significant difference observed between the groups. The mean lingual microleakage score of group I is 2.2±0.83, group II is 1.5±0.54, group III is 1.14±0.37, group IV is 1.66±1.21 and group V is 1.80±1.09. Thus found, no significant difference observed between the groups. Table 3 showed, highly significance for group I, II & III at labial side while on the other hand, group II, III and V showed highly significance at lingual side.

## Discussion:

Orthodontic treatment (OT) involves the use of braces, bands, and wires to move teeth into better positions within the jaw. This procedure is crucial not only for aesthetic purposes but also to enhance function and overall oral health (OH). It primarily involves attaching brackets and bands and applying the desired forces by inserting wires into the bracket slots [5]. The major drawback seen with FO appliances is biofilm retention and plaque accumulation that eventually leads to high oral microbial load (MB-L). As a result, it can lead to white spot lesions, PD problems, enamel-decalcification that can damage intact enamel surface (IES) [5]. Hence, to prevent this harm to tooth structure integrity (TSI), various oral prophylaxis methods have been introduced for instance, chemical methods such as 0.2% CHX mouthwash, electric brushes (E-B), professionally teeth cleaning methods include use of Ultrasonography scalers. However, caries still remains to be the most prevalent condition amongst patients undergoing Facilitated Orthodontic Therapy (FOT). According to the literature, the bacteria involved for dental caries and PD-D are SM-S and LB-S [6]. Over the past two decades, GIC have gained popularity for band cementation (BD-CM) due to their capacity to adhere well to enamel and metal, as well as their ability to release and absorb fluoride. This cement has the inherit property of anti-cariogenic (A-CG) and antimicrobial activity by the release of F ions that helps in restraining the bacterial growth (B-G). Although having superior properties compared to most of the D-CM materials, GIC still presents with poor antimicrobial property in an aqueous environment and marginal seal quality [6]. Nanomaterials were introduced and widely used in dentistry to enhance the properties of GIC. One such NP widely used is Cu-NP. For a long time, Cu has shown antimicrobial effects [6]. Gutiérrez *et al.* found that addition of Cu-NP copper nanoparticles did not affect several mechanical properties tested and higher concentration of Cu-NP produced ADI that are more resistant to ML. The Cu-NP significantly increased antimicrobial activity and also enhances the BS on the teeth interface, therefore inhibiting micro-leakage [7]. In our study, N-AC (10% & 15%) & Cu-NP (2% and 3%) by weight were incorporated into GIC at different concentration to enhance its antimicrobial property and reduce micro-leakage following BD-CM. 5 bottles of GIC powder, each containing 10 grams, were obtained and divided into 5 groups. Group I served as the control group with conventional GIC. Group II consisted of GIC + 2% Cu-NP and 10% N-AC. Group III involved GIC + 2% Cu-NP & 15% N-AC. Group IV contained + 3% Cu-NP & 10% N-AC, while Group V included GIC + 3% Cu-NP & 15% N-AC. The NP and N-AC were accurately weighed using an analytical scale for incorporation. Then the powder was placed into amalgam capsules (AM-CP) and submitted to the action of amalgamator (AMG) with vibration for 6 seconds. Moreover, SM-S was obtained and cultured on selective media to establish a viable stock. Next, SMHA was carefully poured into Petri plates (PP), ensuring a consistent depth(C-DP), and left to solidify. Afterward, 5-6 colonies of SM-S were meticulously selected for sensitivity testing (ST). These colonies were inoculated into a nutrient-rich broth (NRB) and placed in an appropriate incubation (ICB) environment for several hours to allow for growth. After an ICB period of 24-48 hours, the BT-C was spread evenly onto MHA-PL and allowed to dry naturally to remove excess moisture. Subsequently, each well was IC with SM-S-C. The PL was then placed in a controlled IB set at the optimal temperature for bacterial growth (BG). Following IB, the plates were carefully examined to observe any ZOI around the wells, indicating the effectiveness of the test substances against BA. DG-CP was used to measure both the specimens and the diameter of the ZOI after 48 hours. Each tooth was sectioned buccolingually into two halves using a stereomicroscope at 20X magnification, and dye penetration along the cement-enamel interfaces was evaluated. Scoring for dye penetration was conducted from both buccal and lingual margins of the bands following the grading system described by Souza *et al.* [8]. The present study shows statistically significant differences (p-value < 0.05) in all the groups except control group *i.e.* group I. However, the values for subsequent groups (Group II, III, and IV) did not adhere to a normal distribution. Group V (GIC incorporated with 3% Cu-NP & 15% N-AC) displayed the highest ZOI, with an average value of 4.11±0.14 which indicates highest concentration of N-AC & Cu-NP has more efficient antimicrobial activity. Whereas, Group IV (GIC incorporated with 3% Cu-NP and 10% N-AC) showed ZOI with a mean value of 3.41 ± 0.524 which indicates the antimicrobial activity less than the group V. There was minimal variation in the antimicrobial effectiveness between group II & III. Our study also found varying levels of antimicrobial activity among different formulations of GIC modified with N-AC and Cu-NP.

Group I exhibited minimal antimicrobial activity with the smallest ZOI (0.1 mm), indicating limited effectiveness. Conversely, Groups II, III, IV & V showed increased antimicrobial activity with mean zone diameters of approximately 2.6 ± 0.16 mm, 2.1 ± 0.13 mm, 3.4 ± 0.52 mm, and 4.1 ± 0.14 mm, respectively. The results indicate that higher concentration of Cu-NP and N-AC corresponded to greater AM efficacy; particularly group V, demonstrating the highest antimicrobial activity. These findings align with previous studies that have shown enhanced antibacterial properties of modified GIC formulations containing similar additives [9]. Significant differences were observed in pairwise comparisons between group II, IV and V (p = 0.004), underscoring the influence of formulation on antimicrobial effectiveness. Overall, the study underscores the potential of modifying GIC with Cu-NP and N-AC to enhance its AM-P, suggesting promising applications in DM aimed at reducing bacterial growth and improving clinical outcomes. Micro-leakage tests were done using DP were conducted across all sample groups. The highest micro-leakage value was found with group I with a score of 2.2 ± 1.09 and the least score was recorded for group III (0.8± 0.37) with p value = (0.17) being highly significant this is might be because of Cu-NP having increased surface area that improves the physio-mechanical, hence more prevention in seepage of oral fluids. Uysal *et al.* [10] examined the degree of micro-leakage at the interfaces of the cement and enamel and between the cement and the band. The results obtained showed that the BD-CM using conventional GIC exhibited notably elevated levels of micro-leakage at both the cement-band (CM-BD) and CEI. The most significant finding was observed in group III for both the labial and lingual surfaces (p=0.00), with a mean micro-leakage score of 0.85 ± 0.37. This suggests that the addition of 2% Cu-NP and 15% N-AC resulted in minimal ML. However, there was no statistical difference observed for group IV (p=0.65) and group V. This lack of difference could be attributed to the possibility that increasing the concentration of N-AC & Cu-NP may not further reduce micro-leakage efficacy [11]. The mean counts of S. mutans, aerobic and anaerobic lactobacilli, and total bacteria surrounding orthodontic bands cemented with Fuji I containing 8wt% nHA were significantly lower compared to those around orthodontic bands cemented with pure Fuji I (P < 0.05). The study concluded that the incorporation of 8wt% nHA into GI cement may improve its antibacterial properties for the cementation of orthodontic bands, reduce the accumulation of cariogenic bacteria and potentially lower the incidence of caries in orthodontic patients [12]. ZMgO nanoparticles made dental cements much more antibacterial, which means they can resist bacterial microleakage and likely secondary caries. It is suggested that ZMgO nanoparticles be used in cements because they have antibacterial properties that make cavities and gum infections less likely to happen again [13]. A positive effect on reducing microleakage bands surrounding orthodontic bands was revealed by 15% nano-HA-modified banding GIC [14].

## Conclusions:

Modified Glass Ionomer Cement (GIC) exhibited the highest zone of inhibition suggesting strong anti-microbial property. Conventional Glass Ionomer Cement showed the highest micro-leakage score; whereas Glass Ionomer Cement + 2% copper nano-particle and 15% N-acetylcysteine demonstrated the lowest value. Therefore, we conclude that, as the concentration of N-AC and Cu-NP increases, anti-microbial efficiency also increases.

## Figures and Tables

**Table 1:** Grading system

**Score 0**	**No dye penetration**
Score 1	Dye penetration to the extent of one occlusal third of the sealant-enamel joint surface
Score 2	Dye penetration to the extent of one middle third of the sealant-enamel joint surface
Score 3	Dye penetration to the extent of one apical third of the sealant-enamel joint surface

**Table 2:** Comparison of am- resistance (r) (zoi- based)

**Tests of Normality**							
	**Group**	**Kolmogorov-Smirnov**			**Shapiro-Wilk**		
		**Statistic**	**df**	**Sig.**	**Statistic**	**df**	**Sig.**
**Zone of inhibition**	Group I	.	6	.	.	6	.
	Group II	0.358	6	0.016 S	0.781	6	0.040 S
	Group III	0.248	6	0.200*	0.789	6	0.047 S
	Group IV	0.401	6	0.003 HS	0.687	6	0.005 HS
	Group V	0.266	6	0.200*	0.917	6	0.485

**Table 3:** Comparison of ml (based on dp)

**Tests of Normality**							
	**Group**	**Kolmogorov-Smirnov**			**Shapiro-Wilk**		
		**Statistic**	**df**	**Sig.**	**Statistic**	**df**	**Sig.**
**Microleakage (labial)**	Group I	0.367	5	0.026 S	0.684	5	0.006S
	Group II	0.492	6	0.000 HS	0.496	6	0.000 HS
	Group III	0.504	7	0.000 HS	0.453	7	0.000 HS
	Group IV	0.31	6	0.074	0.805	6	0.065
	Group V	0.349	5	0.046 S	0.771	5	0.046 S
**Microleakage (lingual)**	Group I	0.231	5	0.200*	0.881	5	0.314
	Group II	0.319	6	0.056	0.683	6	0.004
	Group III	0.504	7	0.000 HS	0.453	7	0.000 HS
	Group IV	0.209	6	0.200*	0.907	6	0.415
	Group V	0.367	5	0.026 S	0.684	5	0.006 HS
